# Professional practice and awareness of child abuse among radiologists and radiologic technologists: results from Saudi Arabia

**DOI:** 10.1007/s00247-022-05561-x

**Published:** 2022-12-15

**Authors:** Nasser M. Alzahrani, Michael Paddock, Annmarie Jeanes, Farag Shuweihdi, Amaka C. Offiah

**Affiliations:** 1 Department of Oncology and Metabolism, University of Sheffield, Damer Street Building, Western Bank, Sheffield, S10 2TH United Kingdom; 2 Diagnostic Radiology Department, College of Applied Medical Sciences, King Abdulaziz University, Jeddah, Saudi Arabia; 3 Medical Imaging Department, Perth Children’s Hospital, Perth, Western Australia Australia; 4 Department of Paediatric Radiology, Leeds Children’s Hospital, Leeds Teaching Hospitals NHS Trust, Leeds, United Kingdom; 5 Leeds Institute of Health Sciences, University of Leeds, Leeds, United Kingdom; 6 Department of Radiology, Sheffield Children’s NHS Foundation Trust, Western Bank, Sheffield, United Kingdom

**Keywords:** Child abuse, Infants, Paediatric radiology, Radiography, Radiologic technologists, Radiologists, Skeletal survey, Survey

## Abstract

**Background:**

The knowledge, awareness and professionalism of health care providers in the field of child protection are crucial in identifying and reporting suspected child abuse. Radiologic technologists and radiologists play a vital role in the diagnosis of suspected physical child abuse.

**Objective:**

To assess current practice, knowledge and awareness of child abuse among radiologic technologists and radiologists in Saudi Arabia.

**Materials and methods:**

We distributed an internet-based questionnaire to radiologic technologists and radiologists working in Saudi Arabia via national radiology societies and social media channels over a 6-week period (27 October to 8 December 2021). Survey questions covered knowledge regarding child abuse, professional practice in radiology departments in Saudi Arabia in cases of suspected physical abuse (SPA), and knowledge of the national legislation and reporting and acting procedures in child abuse.

**Results:**

A total of 315 respondents (224 radiologic technologists and 91 radiologists) participated in this study. The median score for knowledge of abuse was higher amongst radiologists (4.8) than radiologic technologists (4.0); *P* < 0.001. In total, 210 (93.8%) radiologic technologists and 61 (67.0%) radiologists reported that there was no protocol (i.e. skeletal survey) at their hospital for imaging children with SPA. Most radiologic technologists had no training in paediatric radiology (165/224, 73.7%) and most radiologists had received no training in evaluating imaging performed for SPA (73/91, 80.2%). More than half of respondents — 131 (58.5%) radiologic technologists and 44 (48.4%) radiologists — were not familiar with the reporting and acting procedures at their hospitals in cases of child abuse.

**Conclusion:**

Although radiologic technologists and radiologists in Saudi Arabia have good knowledge and awareness of child abuse in general, they lack specific knowledge of the reporting and acting procedures at their hospitals in cases of suspected child abuse. National imaging guidelines and training courses are needed to develop appropriate skills in the recognition, imaging and reporting of SPA in infants and young children in Saudi Arabia.

**Supplementary Information:**

The online version contains supplementary material available at 10.1007/s00247-022-05561-x.

## Introduction

Child abuse, a significant worldwide problem with serious long-term consequences, has been defined by the World Health Organization as “every kind of physical, sexual, emotional abuse, neglect or negligent treatment, commercial or other exploitation resulting in actual or potential harm to the child’s health, survival, development or dignity in the context of a relationship of responsibility, trust or power” [1]. Globally, it has been estimated that 1 billion children experience abuse over the course of 1 year [2], which might be underestimated given that abuse can go unreported or be incorrectly reported as other causes of death, such as falls and drowning.

Child abuse recognition is challenging and complex, more so in developing countries because of the perceived lack of awareness of child abuse and lack of institutional legislation on child protection, in addition to societal influences and cultural contexts. In Saudi Arabia, child abuse was not well reported until 1990 because of a lack of awareness amongst health care professionals alongside the belief that reporting might affect reporter job security [3, 4]. In recent decades, Saudi Arabia has worked to improve child welfare by ratifying the United Nations Convention on the Rights of the Child (CRC) [5] and, in 2004, by establishing the National Family Safety Program (NFSP), which is concerned with the prevention of child abuse, neglect and domestic violence [3, 5] and which elaborates on the role of the health care system and the regulations around cases of child abuse. At the time of this writing, the National Health Council, the highest health services authority in Saudi Arabia, had accredited 62 major hospitals as hospital-based child protection centres — hospitals with child protection units that deal with issues relating to safeguarding children and young people [6]. Moreover, health care professionals in Saudi Arabia are now legally obliged to report child abuse [3, 7].

Although paediatricians and general practitioners play a vital role in identifying suspected child abuse, the diagnosis is not straightforward and requires a multidisciplinary team approach. Diagnostic medical imaging is instrumental in the diagnosis of suspected physical abuse (SPA) in infants and young children. As the first health care professionals to view any radiographic imaging obtained, radiologic technologists (health care professionals who are trained and qualified to perform diagnostic imaging examinations using various imaging modalities, also known as radiographers or technicians) play a crucial role in raising the suspicion of SPA to colleagues and radiologists through the identification of the suspicious radiographic findings such as rib and metaphyseal fractures, multiple fractures at different stages of healing [8], and potentially inappropriate interactions between caregivers and the child [9]. Clinical radiologists (specialist medical doctors who are trained and qualified to interpret and report upon medical imaging to diagnose injuries and diseases) play a pivotal role in SPA through the identification and reporting of acute and healing fractures when physical abuse is not suspected or when injuries are not clinically apparent (occult) or disclosed (e.g., intracranial haemorrhage) [10, 11].

Knowledge, awareness and adequate training of all health care professionals regarding child abuse are crucial to the identification and reporting of suspected cases. Whilst a few studies in the published literature have assessed the knowledge and awareness of interns, medical students, paediatricians and general practitioners in suspected child abuse in Saudi Arabia [4, 7, 12–14], they report that paediatricians [7, 12] and general practitioners [13] have a “satisfactory” knowledge of child abuse. To the best of the authors’ knowledge, a study assessing current practice, knowledge and awareness of radiologists and radiologic technologists in relation to child abuse in Saudi Arabia has not been performed.

## Materials and methods

We obtained ethics approval from the University of Sheffield Research Ethics Committee before commencing data collection.

### Survey development

We created an online survey using the Google Forms web-based platform (Mountain View, CA). There were two groups of study participants: radiologic technologists and radiologists. The survey included 22 open and 26 closed questions for radiologic technologists (Online Supplementary Material 1) and radiologists (Online Supplementary Material 2), respectively. All responses were anonymised. No personal identifiable information was collected.

Prior to conducting the survey, we undertook a pilot study with seven radiologic technologists and five radiologists and made minor changes based on this initial participant feedback (regarding question wording). The survey comprised four sections. The first section related to demographic and job information. The second assessed child abuse knowledge, including the recognition of abusive acts towards children, risk factors for child abuse [15, 16] and comprehension of the various terms used when describing physical child abuse. The third section of the survey investigated professional practice in dealing with SPA in radiology departments in Saudi Arabia. The final section assessed knowledge regarding national legislation and reporting procedures and actions in child abuse.

### Survey distribution

We distributed the survey to the membership of national radiology societies in Saudi Arabia, including the Saudi Society of Medical Radiologic Technology (SSMRT) and the Radiological Society of Saudi Arabia (RSSA), via email and social media channels. Additionally, the Saudi Commission for Health Specialties (SCFHS), a national official scientific commission that regulates health care–related practices in Saudi Arabia, sent a participation email invitation to all radiologic technologists and radiologists registered on the SCFHS database. Furthermore, the survey was circulated via the professional network of the first author (snowball sampling) and social media channels (Twitter and established WhatsApp and Telegram groups of radiologic technologists and radiologists working in Saudi Arabia). We collected responses over a 6-week period (27 October to 8 December 2021), sending two reminder emails/messages at fortnightly intervals to maximise the response rate.

### Statistical analysis

Statistical analyses were conducted using SPSS Statistics (v. 27; IBM, Armonk, NY) and R software (v. 4.1.3; The R Foundation, Vienna, Austria) to visualise the data for the 5-point questions. Categorical variables were expressed as counts and percentages. For between comparisons (i.e. radiologic technologists versus radiologists), we used the Mann–Whitney *U* test for ordinal variables and the chi-square test for percentages. *P* < 0.05 was significant.

## Results

### Respondent demographics

Of the 315 respondents, 224 (71.1%) were radiologic technologists and 91 (28.9%) were radiologists; those from Saudi Arabia (radiologic technologists, 193/224, 86.2%; radiologists 56/91, 61.5%) were represented more than other nationalities. Most participants worked at the Ministry of Health (radiologic technologists, 109/224, 48.7%; radiologists, 51/91, 56.0%; Online Supplementary Material 3). More than half of the radiologic technologists and radiologists worked at general hospitals (142/224, 63.4%, and 56/91, 61.5%, respectively). Most respondents were from the Western province (radiologic technologists, 37.9%, 85/224; radiologists, 48.4%, 44/91), followed by the central province of Saudi Arabia (radiologic technologists, 28.1%, 63/224; radiologists, 18.7%, 17/91).

Most radiologic technologists had completed an undergraduate bachelor’s degree as their highest level of educational attainment (142/224, 63.4%), with 34.4% (77/224) reporting less than 5 years of clinical experience and 38.4% (86/224) reporting 5–10 years of experience. Most radiologic technologists worked in a general department (133/224, 59.4%), followed by CT (34/224, 15.2%), US (21/224, 9.4%), MRI (19/224, 8.5%) and nuclear medicine (7/224, 3.1%).

Consultant radiologists (medical doctors who have completed a postgraduate specialist radiology training programme, which can include subsequent further specialist clinical training in the form of a postgraduate fellowship, allowing them to be registered as a specialist medical practitioner in their home country, with at least 3–5 years post completion of a specialist training programme) represented more than half of the radiologist respondents (52/91, 57.1%). This group was followed by specialist radiologists (medical doctors who have completed a specialist radiology training programme but have not completed the required 3–5 years of experience post specialist training and thus are not yet consultants) at 35/91 (38.5%) and residents at 4/91 (4.4%). Radiologists’ reported years of experience were less than 5 years (5/91, 5.5%), 5–10 years (33/91, 36.3%), 11–15 years (32/91, 35.2%) and more than 15 years (21/91, 23.1%). Consultant paediatric radiologists (consultant radiologists with a special interest/followship in paediatric radiology) comprised 17.6% (16/91) of the sample, of whom 62.5% (10/16) had 5–10 years of experience. Respondent demographic characteristics and job information are presented in Table 1. Unlike the United Kingdom (UK) and the United States, radiologists in Saudi Arabia need to work for 3–5 years after completing their specialist training/fellowship to become registered as a consultant on the national register. Radiologists can complete the specialist training either overseas or locally (i.e. Saudi Board of Radiology for 4 years).

**Table 1:** Demographic and professional characteristics of participants (*n* = 315)

Variables	Radiologic technologists (*n* = 224)		Radiologists (*n* = 91)	
Nationality	*n* (%)		*n* (%)	
Saudi Non-Saudi	193 (86.2) 31 (13.8)		56 (61.5) 35 (38.5)	
Type of hospital	*n* (%)		*n* (%)	
Medical city/speciality hospital General hospital Primary health care centres	60 (26.8) 142 (63.4) 22 (9.8)		34 (37.4) 56 (61.5) 1 (1.1)	
Region of work	*n* (%)		*n* (%)	
Central province Western province Eastern province Southern province Northern province	63 (28.1) 85 (37.9) 21 (9.4) 40 (17.9) 15 (6.7)		17 (18.7) 44 (48.4) 10 (11.0) 14 (15.4) 6 (6.6)	
	Qualification	*n* (%)	Job title	*n* (%)
	Diploma Bachelor Master PhD or other training certificates (i.e. Board)	55 (24.6) 142 (63.4) 18 (8.0) 9 (4.0)	Resident radiologist Specialist radiologist Consultant radiologist	4 (4.4) 35 (38.5) 52 (57.1)
	Imaging unit mostly covered	*n* (%)	Specialisation	*n* (%)
	General radiography MRI Fluoroscopy Dental radiography CT Mammography Nuclear medicine Ultrasound DEXA Interventional radiology	133 (59.4) 19 (8.5) 0 2 (0.9) 34 (15.2) 2 (0.9) 7 (3.1) 21 (9.4) 1 (0.4) 5 (2.2)	Paediatric radiologist Non-paediatric radiologist	16 (17.6) 75 (82.4)
Years of experience	*n* (%)		*n* (%)	
Less than 5 5–10 11–15 More than 15	77 (34.4) 86 (38.4) 40 (17.9) 21 (9.4)		5 (5.5) 33 (36.3) 32 (35.2) 21 (23.1)	
			Years of experience as paediatric radiologist	*n* = 16 (%)
			Less than 5 5–10 11–15 More than 15	2 (12.5) 10 (62.5) 2 (12.5) 2 (12.5)

*CT* computed tomography, *DEXA* dual-energy X-ray absorptiometry, *MRI* magnetic resonance imaging

### Knowledge regarding child abuse

**Fig. 1:**
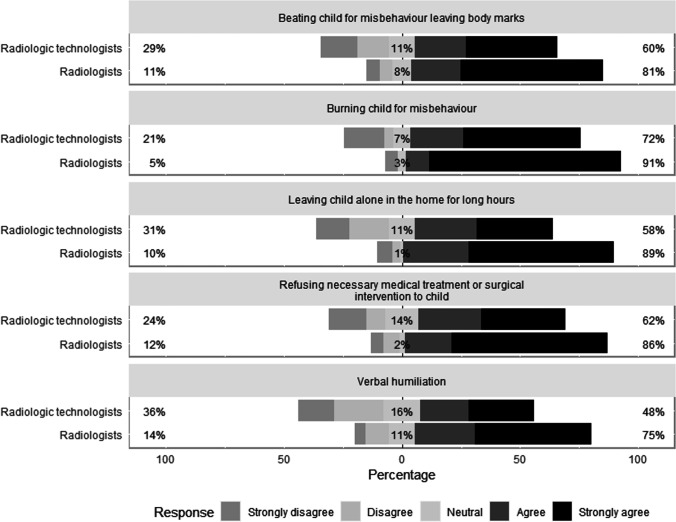
Level of agreement regarding five proposed forms of child abuse. The percentage of neutral responses is indicated on each bar with the total percentage of disagreement (strongly disagree or disagree) noted at left and the total percentage of agreement (strongly agree or agree) noted at right

We assessed several aspects of knowledge regarding child abuse: recognition of abusive acts towards children, recognition of risk factors for child abuse, and understanding of the various terms relating to physical child abuse. The survey asked participants their thoughts about five abusive acts towards children and their responses are presented in Fig. 1. Although both groups had good knowledge regarding the acts of child abuse, radiologists tended to demonstrate a higher score in all five items compared to radiologic technologists, with median scores of 4.8 and 4.0, respectively (*P* < 0.001).

**Fig. 2:**
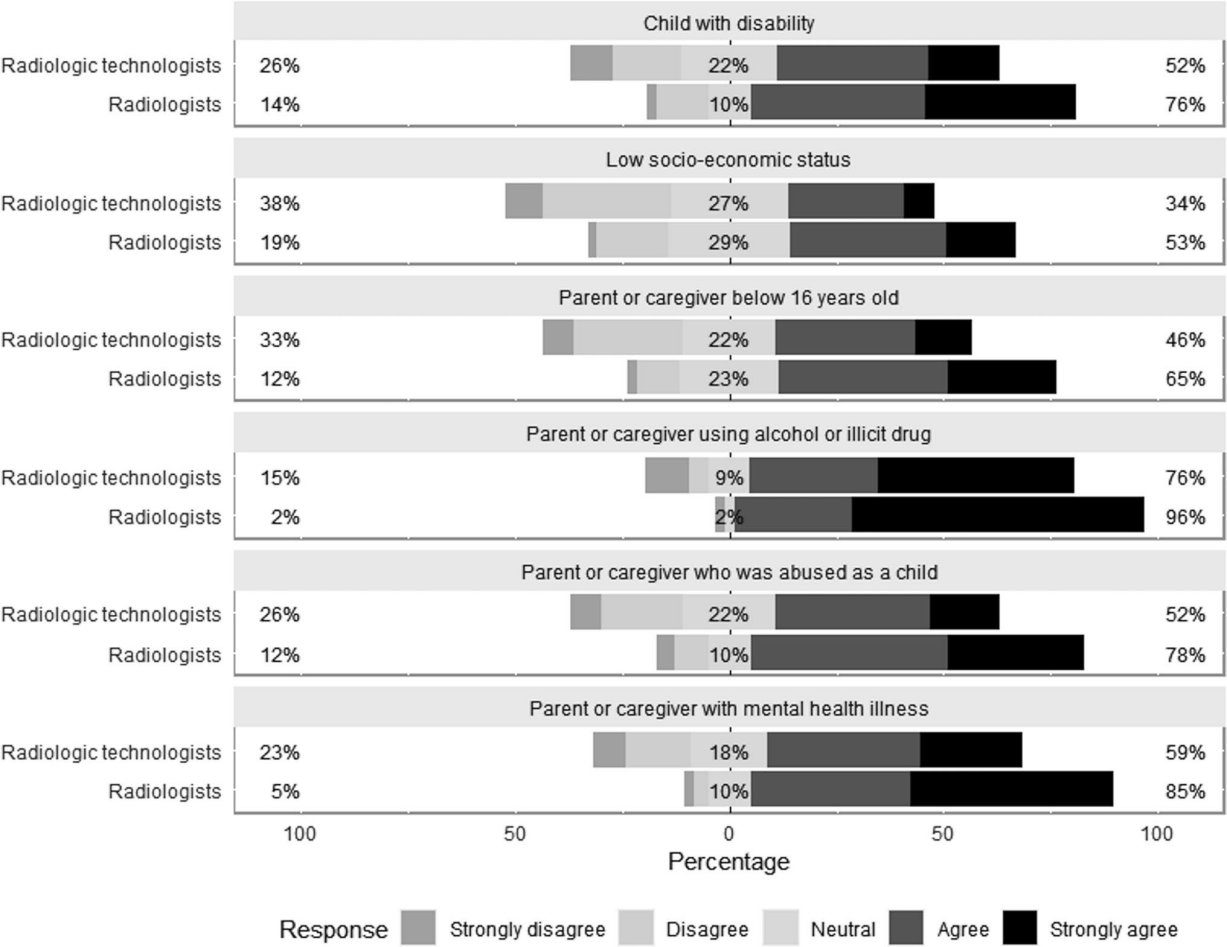
Level of agreement regarding six proposed risk factors for child abuse. The percentage of neutral responses is indicated on each bar with the total percentage of disagreement (strongly disagree or disagree) noted at left and the total percentage of agreement (strongly agree or agree) noted at right

When asked about risk factors for child abuse, the responses varied among radiologic technologists and radiologists (Fig. 2). More than half of radiologists believed that low socioeconomic status is a risk factor for child abuse (48/91, 53.0%) compared to 34.0% (77/224) of radiologic technologists. Similarly, 65.0% (59/91) of radiologists compared to 46.0% (102/224) of radiologic technologists believed that having a parent or caregiver younger than 16 years is a risk factor for child abuse. Radiologists had a greater knowledge of these risk factors compared to radiologic technologists, with median scores of 4.0 and 3.5, respectively (*P* < 0.001).

With regard to the understanding of the term *child physical abuse* and its synonyms (inflicted injury and non-accidental injury), radiologic technologists and radiologists demonstrated good and excellent understanding of the term, respectively (154/224, 68.8% for technologists and 85/91, 93.4% for radiologists), with radiologists having a higher median score compared to radiologic technologists (5.0 and 4.0, respectively; *P* < 0.001; Online Supplementary Material 3).

### The radiologic practice of imaging children with suspected physical abuse

Of the 335 participants, 92.0% (206/224) of radiologic technologists and 83.5% (76/91) of radiologists reported that their practice covers paediatric patients (Online Supplementary Material 3). Of those, 66.9% (138/206) of radiologic technologists and 56.6% (43/76) of radiologists estimated that they image/report on 1–10 cases of SPA annually (Online Supplementary Material 3).

With regard to protocols, 93.8% (210/224) of radiologic technologists and 67.0% (61/91) of radiologists reported that no skeletal survey protocols existed at their hospital for imaging children for SPA (Table 2). We found great variation in the protocols for imaging children for SPA in Saudi Arabia (Online Supplementary Material 3). Most radiologic technologists (193/224, 86.2%) and more than half of radiologists (55/91, 60.4%) had no awareness of the published international guidelines for imaging children with SPA (Table 2).

**Table 2:** Participants’ responses to professional practice regarding imaging children with suspected physical abuse

Questions	Radiologic technologists (*n* = 224)		Radiologists (*n* = 91)	
	Yes	No	Yes	No
	*n* (%)	*n* (%)	*n* (%)	*n* (%)
Is there an imaging protocol (i.e. skeletal survey) at your hospital for children younger than 2 years with suspected physical abuse?	14 (6.3)	210 (93.8)	30 (33.0)	61 (67.0)
Are you aware of the international guidelines for imaging children with suspected physical abuse?	31 (13.8)	193 (86.2)	36 (39.6)	55 (60.4)
Have you received training (i.e. courses, workshops, workplace mentoring, etc.) in paediatric radiology?	59 (26.3)	165 (73.7)	N/A	
Have you received training (i.e. courses, workshops, etc.) in evaluating injury in children related to suspected physical abuse?	N/A		18 (19.8)	73 (80.2)
Is there a paediatric radiologist at your hospital?	N/A		40 (44.0)	51 (56.0)
Is there a named radiologist at your hospital to report cases with suspected abuse?	N/A		12 (13.2)	79 (86.8)
Are the radiologic images of suspected child abuse reported by at least 2 radiologists at your hospital?	N/A		11 (12.1)	80 (87.9)

*N/A* not applicable

Availability of paediatric radiologists was lacking in many hospitals: more than half of radiologists (51/91, 56.0%) reported having no paediatric radiologist at their workplace. Most radiologists said that no radiologist was assigned to report radiologic imaging performed for SPA (79/91, 86.8%) and that there was no double reporting (by at least two radiologists) of such imaging studies at their hospitals (80/91, 87.9%) (Table 2).

**Fig. 3:**
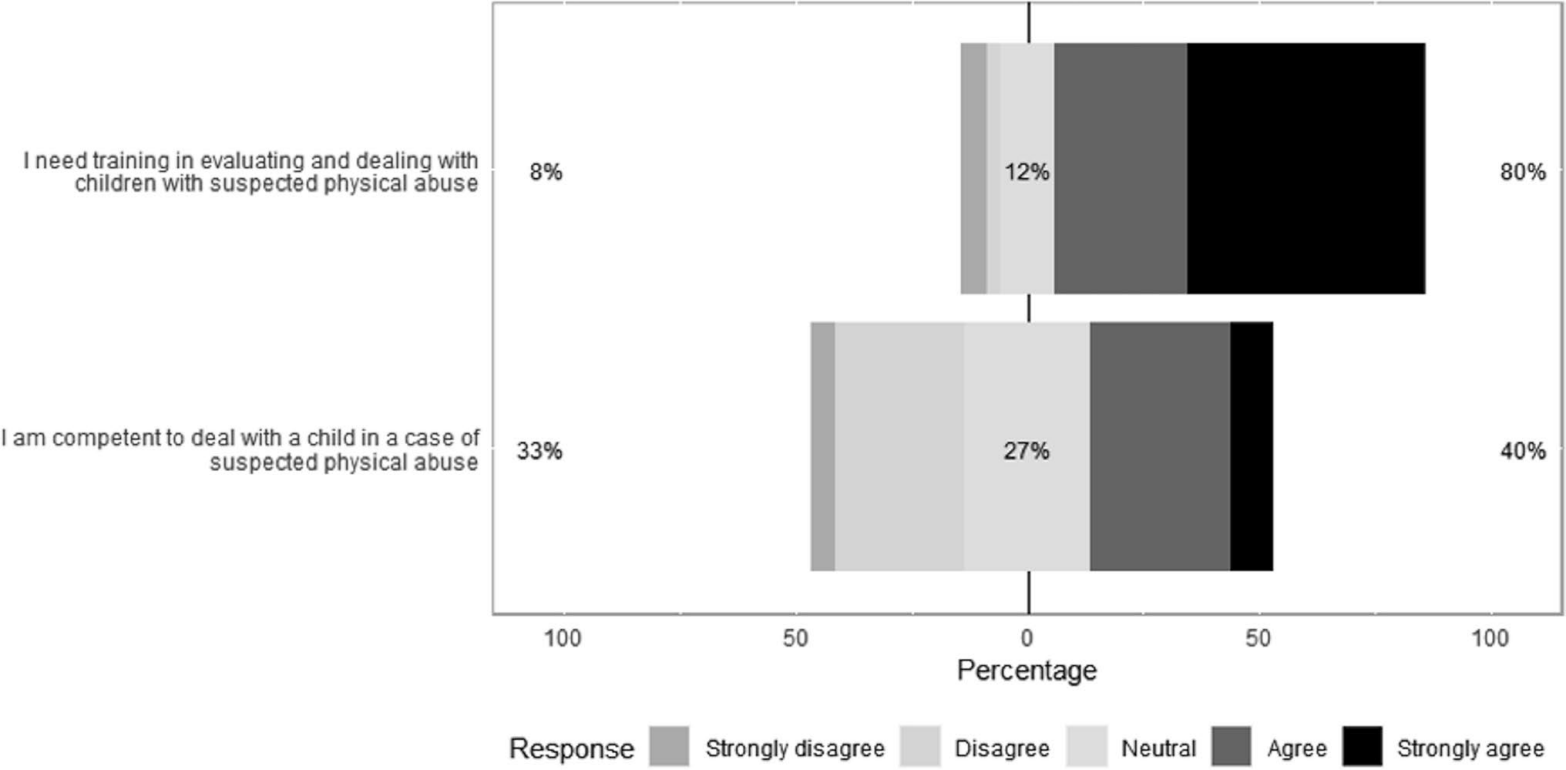
Radiologic technologists’ opinions regarding their training needs and their confidence in evaluating and dealing with cases of suspected physical abuse. The percentage of neutral responses is indicated on each bar with the total percentage of disagreement (strongly disagree or disagree) noted at left and the total percentage of agreement (strongly agree or agree) noted at right

On the question of training, most radiologic technologists (165/224, 73.7%) had no training in paediatric radiology (Table 2). Of those radiologic technologists who had received training in paediatric radiology, only 32.2% (19/59) stated that their training discussed injuries relating to SPA (Online Supplementary Material 3). Furthermore, it was much more common for radiologic technologists to attend the national training format in Saudi Arabia (i.e. courses, workshops or conferences) related to paediatric radiology (37/59, 62.7%) as opposed to the international training format (4/59, 6.8%; Online Supplementary Material 3). Finally, 33.5% (75/224) of radiologic technologists responded that they thought they were not competent to be involved with cases of SPA, with most indicating that they required training for such cases (179/224, 80.0%) (Fig. 3).

**Fig. 4:**
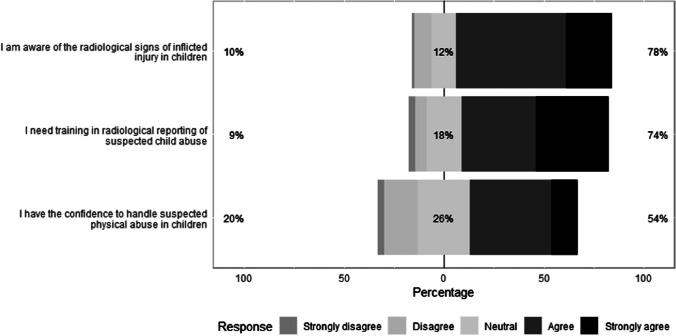
Radiologists’ opinions regarding recognising the radiologic signs, their training needs and confidence in the radiologic reporting of cases of suspected physical abuse. The percentage of neutral responses is indicated on each bar with the total percentage of disagreement (strongly disagree or disagree) noted at left and the total percentage of agreement (strongly agree or agree) noted at right

Among radiologists, most had received no training in evaluating radiologic imaging in children performed for SPA (73/91, 80.2%). Of those radiologists who had received such training, it was slightly more common that they had undertaken the international training format (i.e. courses, workshops or conferences; 8/18, 44.4%) as opposed to the national training format (6/18, 33.3%; Online Supplementary Material 3). Most radiologists were aware of the radiologic patterns of inflicted injury in children (71/91, 78.0%) and more than half of radiologists stated that they were confident to report radiologic imaging performed for SPA (49/91, 54.0%). However, most radiologists indicated that they required (further) training (67/91, 74.0%) (Fig. 4).

### Knowledge of national legislation and reporting and acting procedures in child abuse

**Fig. 5:**
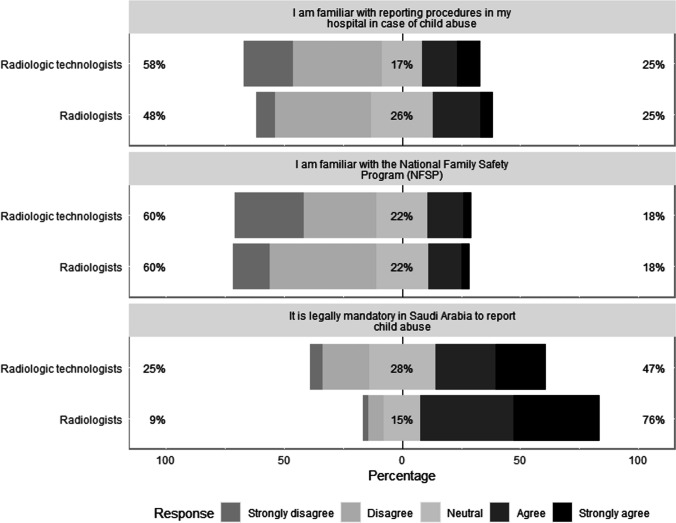
Level of agreement among responders regarding their knowledge of the national law and programs for child abuse and reporting procedures and actions at their hospitals in suspected child abuse cases. The percentage of neutral responses is indicated on each bar with the total percentage of disagreement (strongly disagree or disagree) noted at left and the total percentage of agreement (strongly agree or agree) noted at right

The survey asked participants about the NFSP and the local reporting procedures at their hospitals in cases of SPA in children. More than half of radiologic technologists and radiologists were unfamiliar with the NFSP (134/224, 60.0%, and 55/91, 60.0%, respectively), and 58.5% (131/224) of radiologic technologists and 48.4% (44/91) of radiologists were also unfamiliar with the reporting and action procedures at their hospitals in cases of SPA (Fig. 5). Radiologists were more familiar with the legal mandate to report child abuse in Saudi Arabia (69/91, 76.0%) than were radiologic technologists (105/224, 47.0%). Both groups had little knowledge regarding the reporting procedures and actions in cases of child abuse (all forms); however, radiologists demonstrated a slightly higher score than radiologic technologists regarding reporting procedures in cases of SPA (median scores 3.0 compared to 2.7, respectively; *P* < 0.002).

## Discussion

This cross-sectional survey assessed the current practice, knowledge and awareness of child abuse amongst radiologists and radiologic technologists in Saudi Arabia. Overall, most survey respondents had a good knowledge of the various forms and risk factors for child abuse. However, 48.4% of radiologists and more than half of radiologic technologists (58.5%) lacked knowledge regarding the reporting procedures in cases of child abuse. Moreover, most radiologic technologists (93.8%) and many radiologists (67.0%) reported no imaging protocols (e.g., radiographic skeletal survey) at their hospital for imaging children with SPA.

Child abuse is a serious global problem with severe consequences for victims, their families and society. In Saudi Arabia, the number of child abuse cases is reported to be increasing annually [6, 17]. The NFSP’s national record for child abuse and neglect reported that 428 and 1,253 children were physically abused in 2020 and 2021, respectively [6, 18]. A comprehensive understanding of the various forms of child abuse and the factors that increase the risk of child abuse is vital for health care professionals to facilitate increased detection and reporting of suspected cases.

The definition of abuse (in general) varies among societies. For example, in the Arab culture, corporal punishment and verbal discipline are likely to be considered societal norms [14]. Several risk factors have been established for child abuse and maltreatment, including but not limited to low socioeconomic status/economic disadvantage, caregivers with mental health or drug issues, young parents, and children with disabilities [15, 19, 20]. Although it is well reported that socioeconomic status is an important risk factor for child abuse in the literature [15], more than one-third (36.0%) of radiologic technologists in this study did not recognise this as a risk factor.

Although child abuse takes various forms (emotional abuse, sexual abuse and neglect), physical abuse might be more well-known given the media attention surrounding high-profile cases. However, the diagnosis of physical child abuse is challenging; children require a thorough physical examination, radiologic imaging, laboratory tests and a social evaluation [20, 21]. Given that children who are physically abused are more likely to have negative lifelong consequences such as behavioural problems and neurological disabilities [22], an early and accurate diagnosis of physical child abuse is paramount to improving child health outcomes and preventing potential future harm.

Medical imaging is a crucial tool in the diagnosis and management of children with SPA. Published guidelines for imaging SPA in children include those from the American College of Radiology and the Society for Pediatric Radiology (ACR-SPR) [23], the Royal College of Radiologists (RCR) and the Society and College of Radiographers (SCoR). The last has been endorsed by the Royal College of Paediatrics and Child Health (RCPCH) in the UK [24] and recognised by the European Society of Paediatric Radiology (ESPR) as the gold standard guidance for imaging children with SPA across Europe [25].

The radiographic skeletal survey, which comprises a series of radiographic images of the entire skeleton [23, 24], is a well-established radiologic examination in the investigation of SPA in children. In our study, 93.8% of radiologic technologists and 67.0% of radiologists reported having no SPA skeletal survey protocol at their place of work. Additionally, 86.2% of radiologic technologists and 60.4% of radiologists were unfamiliar with any published guidelines for SPA imaging. It is important that radiology departments have a clear imaging protocol in place for imaging children with SPA; the previously mentioned guidelines [23, 24] serve as exemplar practice and our findings indicate that radiology departments in Saudi Arabia should endorse and utilise one of these imaging guidelines as evidence-based best practice.

Radiologists have a pivotal role in assessing children investigated for SPA by differentiating traumatic injuries from normal variants or other pathologies, recognising features that might indicate underlying metabolic and inherited conditions, and suggesting the most likely time frame and mechanisms for injuries; further, radiologists sometimes play a key role in subsequent legal proceedings [10, 11, 26]. The role of radiologists in SPA is rendered less effective if suspicions regarding physical abuse are not promptly and appropriately communicated to the relevant clinical team. In Saudi Arabia, the child protection team includes a paediatric physician, a psychologist and professionals from social services and a legal department [3]. We recommend that a radiologist also be considered as a key member of the child protection team to facilitate communication of important radiologic findings to aid in clinical and child-protection decision-making.

In the context of evaluating children with SPA, radiology departments should have at least one dedicated paediatric radiologist (or radiologist trained in paediatric imaging) who is designated (and trained) to report skeletal survey imaging; ideally two experienced reporters are required to facilitate independent double reporting [24]. Paediatric-trained radiologists could support and train colleagues in their department to increase the number of available radiologists to double-report such examinations. In the UK, 61.0% of radiology departments have a designated radiologist to report the skeletal survey, and 62.0% of radiology departments reported that the skeletal survey is double-reported by radiologists [26]. In this study, 51.0%, 79.0% and 80.0% of radiologists reported no paediatric radiologist, no designated radiologist to report the skeletal survey, and no double reporting of skeletal surveys, respectively, at their centres.

Unlike general radiologists, paediatric radiologists have more experience and a greater familiarity with normal paediatric bone development, normal variants in children and the radiographic appearance of fracture patterns in inflicted injury. However, paediatric radiology is a relatively small radiologic subspeciality, and there is an international shortage of paediatric radiologists [27–29]. As a consequence, not all hospitals have direct access to a trained paediatric radiologist. We advise that such centres forge links/networks with paediatric radiologists to facilitate access to timely reports on SPA imaging, which in turn could facilitate quicker decision-making. Such a system would also promote continuing professional development in paediatric radiology and SPA imaging. We also recommend involving experienced musculoskeletal radiologists and neuroradiologists to report brain and spine imaging performed for SPA.

In Saudi Arabia, it is necessary to improve not only the practice of paediatric radiography and radiology, particularly in the evaluation of SPA in children, but also the education of relevant professionals on this topic. In Saudi Arabia, the clinical radiology speciality training curriculum includes only a 4-month rotation in paediatric radiology out of the 4-year training program, with a single lecture on fracture patterns in abused children [30]. Trainees enrolled in a UK clinical radiology speciality program usually complete at least a 3-month rotation in paediatric radiology during their 5-year training program [31]. The RCR speciality training curriculum states that trainees are expected to have appropriate skills in interpreting images of inflicted injuries in children and that “examples of” imaging procedures in which all radiology trainees will develop skills to level 4 (fully independent practice) … include accidental and non-accidental injury in children” [31]. Although didactic lectures are important in training programs, the practice of interpreting cases at the workstation with an experienced paediatric radiologist is essential to training. Additionally, discussion with the clinical teams regarding the proffered history and mechanism of injury is of critical importance to contextualising any radiologic findings.

In clinical practice, double reporting is recommended for radiologic studies with elevated risk [32, 33], with the second read improving confidence for less experienced radiologists and offering an improvement in diagnostic accuracy and consistency [32, 34]. Arguably, the skeletal survey performed in cases of SPA could be considered one of the highest-risk examinations performed given the impact of any positive (or negative) findings on the accurate and timely diagnosis of SPA. Moreover, the radiologic report can be critical to legal proceedings, further signifying not only its clinical importance but its utility in the forensic and medicolegal arena.

As health care professionals in Saudi Arabia, radiologic technologists are mandated to alert the relevant authorities to cases of SPA whilst maintaining patient confidentiality and professionalism. In cases of SPA, the role of the radiologic technologist is to produce high-quality diagnostic images using an evidence-based (and reproducible) skeletal survey protocol with careful documentation of the examination, including the time; who was present; and the type, number and dose of projections obtained [24, 35]. The aforementioned guidelines [23, 24] for imaging children with SPA recommend that radiologic technologists who are trained in paediatric imaging perform the skeletal survey. In our study, fewer than half of radiologic technologists (40.0%) reported that they were competent in imaging children in cases of SPA, which might reflect a lack of education and training.

There is a national reporting centre in Saudi Arabia [6] and health care professionals are mandated by law to report suspected abuse to child protective services [3]. In this study, 76.0% of radiologists and 47.0% of radiologic technologists were aware of the existing law in Saudi Arabia relating to the mandated reporting of all types of child abuse. However, few participants (25.0% in each group) were familiar with their workplace’s reporting *procedures* for suspected child abuse. This result is consistent with the results of other surveys conducted among health professionals in Saudi Arabia concerning suspected child abuse [12, 13]. The reason for the lack of knowledge regarding reporting procedures, which we found in up to 60.0% of radiologists and radiologic technologists in our study, could be a lack of training and awareness of the NFSP. We hope the survey increased knowledge of the NFSP among radiologic technologists and radiologists who participated and that any lack of knowledge could be further remedied with ongoing promotion of the program on a national scale.

Training health care professionals in recognising, evaluating and reporting child abuse is central for improving and promoting the health and welfare of children. In this study, most participants had not received training to deal with or evaluate cases of SPA, with many respondents reporting the need for (further) training in such cases. About 73% of radiologic technologists in this study had not received training in paediatric radiology and 64% of radiologic technologists had not received training related to imaging children with SPA. The results of our study correlate with a study conducted in Nigeria, which demonstrated that 49% and 65% of radiologic technologists had not received training in paediatric radiology and imaging children with SPA, respectively [9]. Improving the education system and establishing training courses in paediatric radiology, with specific reference to SPA, are necessary to enable radiologic technologists and radiologists to engage in this important facet of their practice whilst working alongside other health care professionals to protect vulnerable children. Furthermore, a dedicated training program for health care professionals in Saudi Arabia is needed to help them recognise cases of SPA and to outline the steps of reporting to the appropriate authorities within the workplace.

This study has several limitations. First, although we distributed the survey via various channels, and included reminders, the findings must be interpreted with caution because of the small number of responders (the overall number of members in the national radiology/radiography societies was not known at the time of the study). The small number of participants could be the result of a lack of interest in research in general, or more specifically in paediatric radiology, where it is well recognised that there is a significant clinical workforce shortage [27, 29, 36]. In addition, email-based contact might have been labelled “junk” by mail servers, increasing the possibility that intended emails were not received or read by recipients. The number of radiologic technologists and radiologists in Saudi Arabia is not small but, unfortunately, we are not able to ascertain the exact number because of the lack of statistical data provided by the Ministry of Health in Saudi Arabia. Second, there is sampling bias caused by the online design of the survey whereby access might have been restricted to only those with email or social media accounts (i.e. Twitter, WhatsApp and Telegram) and those working in Saudi Arabia radiology departments. Furthermore, there might be a response bias, with responses potentially being biased by personal experience or opinions regarding child abuse, or by cultural background/understanding of child abuse from the non-Saudi radiologic technologists and radiologists, who represented 13.8% and 38.5% of respondents, respectively. Additionally, although most participating radiologic technologists were primarily working with modalities commonly used for imaging children with SPA, such as general radiography (59.4%), CT (15.2%) and MRI (8.5%), a few technologists were more involved with other modalities that are far less likely (US, 9.4%; nuclear medicine, 3.1%; and dental radiography, 0.9%) or never (e.g., interventional radiology, 2.2%; and mammography, 0.9% of respondents) used in imaging children with SPA. However, the results of this study still provide a valuable insight into the current practice, knowledge and awareness of child abuse amongst radiologists and radiologic technologists in Saudi Arabia. The final limitation is that the study was restricted to Saudi Arabia and might therefore be of limited relevance to readers from other countries. However, it could represent a similar lack of awareness of child physical abuse in other lower-income countries in Africa and the Middle East. Our results serve as a baseline to which other countries can compare their findings, and with which Saudi Arabia can compare itself in future studies. The ESPR and SPR are significantly involved in outreach work through the World Federation of Pediatric Imaging, and perhaps through them future international studies can be conducted. Importantly, child abuse is a worldwide problem and therefore dissemination of such a study in internationally recognised journals should lead to the sharing of knowledge on a wider international scale, increasing the awareness of health care professionals of child protection and reminding them of the role of medical imaging in diagnosing SPA.

## Conclusion

Radiologic technologists and radiologists, alongside all health care professionals who work with or alongside children, play an integral role in the diagnosis and management of children suspected of having been physically abused. This study has demonstrated that overall knowledge and awareness of child abuse amongst radiologists and radiologic technologists in Saudi Arabia is good; however, they lack specific knowledge regarding the reporting and acting procedures at their hospitals in cases of suspected child abuse. Moreover, there is a lack of standardised protocols and radiologic reporting expertise for cases of suspected child abuse. This study highlights a clear need for national imaging guidelines and training programs relating to the imaging and radiologic reporting of suspected physical abuse in infants and young children in Saudi Arabia.

## Supplementary Information

Below is the link to the electronic supplementary material.

Supplementary file1 (PDF 157 KB)

Supplementary file2 (PDF 159 KB)

Supplementary file3 (DOCX 28.0 KB)

## References

[1] World Health Organization (2021) Child maltreatment. https://www.who.int/news-room/fact-sheets/detail/child-maltreatment. Accessed 21 Feb 2022

[2] Hillis S, Mercy J, Amobi A, Kress H (2016). Global prevalence of past-year violence against children: a systematic review and minimum estimates. Pediatrics.

[3] Al Eissa M, Almuneef M (2010). Child abuse and neglect in Saudi Arabia: journey of recognition to implementation of national prevention strategies. Child Abuse Negl.

[4] Alanazi SS, Althaqib AN, Albeladi KE (2021). Child abuse and neglect awareness between knowledge, perception, and reporting among interns and medical students of Majmaah University. IJMDC.

[5] Almuneef M, Al-Eissa M (2011). Preventing child abuse and neglect in Saudi Arabia: are we ready?. Ann Saudi Med.

[6] National Family Safety Program (2020) National record for child abuse and neglect. https://nfsp.org.sa/ar/projects/NationalRecord/Documents/nfsr_2020_report.pdf. Accessed 5 Mar 2022

[7] Habib HS (2012). Pediatrician knowledge, perception, and experience on child abuse and neglect in Saudi Arabia. Ann Saudi Med.

[8] Doyle E, Vuong R (2020). A literature review of ‘best practice’ for radiographers when imaging suspected non-accidental injury or physical abuse of children in Australia and New Zealand. J Forensic Radiol Imaging.

[9] Ewuzie O (2021). Nigerian radiographers and non-accidental injury in children. Int J Med Health Dev.

[10] Offiah AC (2012). Radiological features of child maltreatment. Paediatr Child Health.

[11] Negus S (2015). The role of radiology in child abuse. Infant J.

[12] Alnasser Y, Albijadi A, Abdullah W (2017). Child maltreatment between knowledge, attitude and beliefs among Saudi pediatricians, pediatric residency trainees and medical students. Ann Med Surg.

[13] Aldukhayel A, Aljarbou E, Alturki FM (2020). Knowledge and attitude regarding child abuse among primary healthcare physicians and interns in Al Qassim. Saudi Arabia Cureus.

[14] Al-Dabaan R, Newton JT, Asimakopoulou K (2014). Knowledge, attitudes, and experience of dentists living in Saudi Arabia toward child abuse and neglect. Saudi Dent J.

[15] Austin AE, Lesak AM, Shanahan ME (2020). Risk and protective factors for child maltreatment: a review. Curr Epidemiol Rep.

[16] Stith SM, Liu T, Davies LC (2009). Risk factors in child maltreatment: a meta-analytic review of the literature. Aggress Violent Behav.

[17] Ghaffar UB, Ahmad M, Faraz A, Ahmad A (2018). A study of child abuse trend in Saudi Arabia — a review update. Indian J Forensic Community Med.

[18] National Family Safety Program (2021) National record for child abuse and neglect. https://nfsp.org.sa/ar/awareness/DocLib/2021%20Report.pdf. Accessed 18 Sep 2022

[19] Doidge JC, Higgins DJ, Delfabbro P, Segal L (2017). Risk factors for child maltreatment in an Australian population-based birth cohort. Child Abuse Negl.

[20] Christian CW, Committee on Child Abuse and Neglect, American Academy of Pediatrics (2015) The evaluation of suspected child physical abuse. Pediatrics 135:e1337–e1354 10.1542/peds.2015-0356 25917988

[21] Blangis F, Allali S, Cohen JF (2021). Variations in guidelines for diagnosis of child physical abuse in high-income countries: a systematic review. JAMA Netw Open.

[22] Gilbert R, Widom CS, Browne K (2009). Burden and consequences of child maltreatment in high-income countries. Lancet.

[23] American College of Radiology, Society for Pediatric Radiology (2021) ACR–SPR practice parameter for the performance and interpretation of skeletal surveys in children. https://www.acr.org/-/media/ACR/Files/Practice-Parameters/Skeletal-Survey.pdf. Accessed 25 Feb 2022

[24] Royal College of Radiologists, Society and College of Radiographers (2017) The radiological investigation of suspected physical abuse in children. https://www.rcr.ac.uk/system/files/publication/field_publication_files/bfcr174_suspected_physical_abuse.pdf. Accessed 25 Feb 2022

[25] Offiah AC, Adamsbaum C, van Rijn RR (2014). ESPR adopts British guidelines for imaging in suspected non-accidental injury as the European standard. Pediatr Radiol.

[26] Leung RS, Nwachuckwu C, Pervaiz A (2009). Are UK radiologists satisfied with the training and support received in suspected child abuse?. Clin Radiol.

[27] Halliday K, Drinkwater K, Howlett DC (2016). Evaluation of paediatric radiology services in hospitals in the UK. Clin Radiol.

[28] Ng K-L, Yazer J, Abdolell M, Brown P (2010). National survey to identify subspecialties at risk for physician shortages in Canadian academic radiology departments. Can Assoc Radiol J.

[29] Farmakis SG, Chertoff JD, Barth RA (2021). Pediatric radiologist workforce shortage: action steps to resolve. J Am Coll Radiol.

[30] Saudi Commission for Health Specialties (2015) Saudi medical imaging curriculum. https://scfhs.org.sa/sites/default/files/2022-02/DIAGNOSTIC%20RADIOLOGY-%20new%20cover2021.pdf. Accessed 16 Mar 2022

[31] Royal College of Radiologists (2021) Clinical radiology specialty training curriculum. https://www.rcr.ac.uk/sites/default/files/clinical_radiology_curriculum_2020.pdf. Accessed 16 Mar 2022

[32] Lauritzen PM, Stavem K, Andersen JG (2016). Double reading of current chest CT examinations: clinical importance of changes to radiology reports. Eur J Radiol.

[33] Marine MB, Forbes-Amrhein MM (2021). Fractures of child abuse. Pediatr Radiol.

[34] Karmazyn B, Miller EM, Lay SE (2017). Double-read of skeletal surveys in suspected non-accidental trauma: what we learned. Pediatr Radiol.

[35] Brown A-M, Henwood SM (1997). Good practice for radiographers in non-accidental injury. Radiography.

[36] Royal College of Radiologists (2020) Clinical radiology UK workforce census 2020 report. https://www.rcr.ac.uk/system/files/publication/field_publication_files/clinical-radiology-uk-workforce-census-2020-report.pdf. Accessed 17 May 2022

