# Supplementary material for: Professional practice and awareness of child abuse among radiologists and radiologic technologists: results from Saudi Arabia
Source: Pediatr Radiol. 2022 Dec 15;53(5):832–43. doi: 10.1007/s00247-022-05561-x (PMC10156848; doi:10.1007/s00247-022-05561-x)
Supplement: Supplementary file 2 — Supplementary file2 (PDF 159 KB) [file 247_2022_5561_MOESM2_ESM.pdf]

Professional Practice and Awareness of Child Abuse Among Radiologists and Radiologic technologists in Saudi Arabia

Dear Participant,

You are invited to participate in a web-based survey on professional practice and awareness of child abuse among radiologists and radiographers in Saudi Arabia. This survey is part of a research project concerning the radiological investigation of suspected physical abuse in children which is being conducted by BLINDED, a BLINDED at the BLINDED, BLINDED, under the primary supervision of BLINDED.

The survey should take no longer than 5 minutes to complete.

Your participation in this survey is voluntary. You may exit the survey without submission at any time and your answers will not be collected. This study has received a favourable opinion by the University Research Ethics Committee.

There are no risks associated with participating in this study. There are no costs or payments associated with your participation in the study. However, your responses may help us learn more about the current practice and knowledge of radiologists and radiographers in Saudi Arabia regarding suspected child abuse.

The survey is anonymous. No identifiable information, such as name, sex, date of birth, contact information or IP address, will be collected. Your survey answers will be sent to a Google Form where the data will be stored in password-protected electronic format. No one will be able to identify you or your answers, and no one will know whether you participated in the study.

If you have any questions about the study, please contact BLINDED via email at BLINDED

If you feel you have not been treated according to the descriptions in this form, or that your rights as a participant in research have not been honoured during the course of this project, or you have any questions, concerns, or complaints that you wish to address to someone other than the investigator, you may contact the University Data Protection Officer at BLINDED

By completing and submitting this survey, you are indicating your consent to participate in the study. Your participation is appreciated.

Many thanks for your time and cooperation.

---

*Required

Demographics

1. Nationality *

Mark only one oval.

- ( ) Saudi
- ( ) Non-Saudi

2. Place of work *

Mark only one oval.

- ( ) Ministry of Health
- ( ) Universities' Hospital
- ( ) National Guard Hospital
- ( ) King Faisal Specialist Hospital & Research Centre
- ( ) Armed Forces Hospital
- ( ) Security Forces Hospital
- ( ) John Hopkins/Aramco Hospital
- ( ) Royal Commission Hospital
- ( ) Private Hospital
- ( ) Other: ____________________

3. What type of hospital do you work in? *

Mark only one oval.

- Medical city / Speciality hospital
- General hospital
- Primary health care centre
- Other: ____________

4. In which province are you working? *

Mark only one oval.

- Riyadh
- Makkah
- Madinah
- Eastern
- Al-Jawf
- Al-Baha
- Qassim
- Aseer
- Tabuk
- Jazan
- Najran
- Northern Borders
- Hail
- Other: ____________

5. What is your job title? *

Mark only one oval.

- Resident radiologist
- Specialist radiologist
- Consultant radiologist

6. If you answered “Resident radiologist” >> what is your stage/year of training?

Mark only one oval.

- 1st year
- 2nd year
- 3rd year
- 4th year

7. How many years' experience do you have as a radiologist? *

Mark only one oval.

- Less than 5 years
- 5 -10 years
- 11-15 years
- More than 15 years

8. Are you a paediatric radiologist? *

Mark only one oval.

- Yes
- No

9. If you answered "Yes", how many years' experience do you have as a paediatric radiologist?

Mark only one oval.

- Less than 5 years
- 5 -10 years
- 11-15 years
- More than 15 years

knowledge about child abuse

All questions relate to children below 2 years of age (i.e., children who have NOT reached their second birthday

If you are using your smartphone, please rotate the screen to landscape

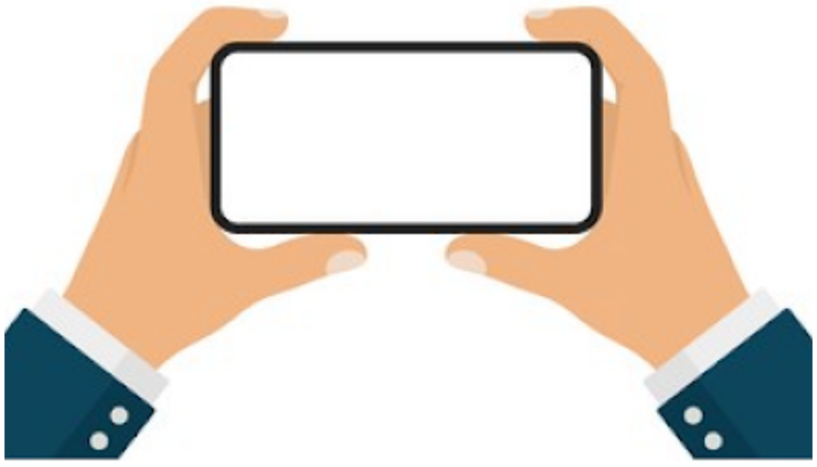

10. Do you consider the following acts as abusive towards children? *

Mark only one oval per row.

	Strongly disagree	Disagree	Neutral	Agree	Strongly agree
**Beating child for misbehaviour leaving body marks**					
**Leaving child alone in the home for long hours**					
**Burning child for misbehaviour**					
**Verbal humiliation**					
**Refusing necessary medical treatment or surgical intervention to child**					

11. Do you consider the following statements as a risk factor for child abuse? *

Mark only one oval per row.

	Strongly disagree	Disagree	Neutral	Agree	Strongly agree
**Child with disability**	◯	◯	◯	◯	◯
**Parent or caregiver below 16 years old**	◯	◯	◯	◯	◯
**Parent or caregiver with mental health illness**	◯	◯	◯	◯	◯
**Low socio-economic status**	◯	◯	◯	◯	◯
**Parent or caregiver who was abused as a child**	◯	◯	◯	◯	◯
**Parent or caregiver using alcohol or illicit drug**	◯	◯	◯	◯	◯

12. To what extent do you agree or disagree with the following statement *

Mark only one oval per row.

	Strongly disagree	Disagree	Neutral	Agree	Strongly agree
**I understand the meaning of the term "inflicted injury" or "physical abuse" or "non-accidental injury" in children?**	◯	◯	◯	◯	◯

Radiological reporting and imaging protocol

13. Does your practice cover paediatric patients (children 0 to 18 years)? *

Mark only one oval.

◯ Yes
◯ No

14. If you answered "Yes", then approximately how many cases of suspected child abuse do you report on per year?

Mark only one oval.

◯ 1-10
◯ 11-20
◯ 21-30
◯ 31-40
◯ 41-50
◯ 51-60
◯ 61-70
◯ 71-80
◯ 81-90
◯ 91-100
◯ > 100

15. Is there an imaging protocol (i.e. skeletal survey) in your hospital for children below 2 years' old with suspected physical abuse? *

Mark only one oval.

◯ Yes
◯ No

16. If you answered "Yes" – what is the protocol?

____

17. Are you aware of the international guidelines for imaging children with suspected physical abuse? *

Mark only one oval.

- ( ) Yes
- ( ) No

18. If you answered "Yes", which guidelines are you aware of?

Tick all that apply.

- [ ] ACR-SPR
- [ ] RCR-SCoR
- [ ] Other: ____________________

19. Have you received training (i.e. courses, workshops, ...etc) in evaluating injury in children related to suspected physical abuse? *

Mark only one oval.

- ( ) Yes
- ( ) No

20. If you answered "Yes", what type of training did you receive?

Tick all that apply.

- [ ] Local course
- [ ] Local workshop
- [ ] Local conference
- [ ] International course
- [ ] International workshop
- [ ] International conference
- [ ] Other: ____________________

21. Is there a paediatric radiologist in your hospital? *

Mark only one oval.

- ( ) Yes
- ( ) No

22. Is there a named radiologist in your hospital to report cases with suspected abuse? *

Mark only one oval.

- ( ) Yes
- ( ) No

23. Are the radiological images of suspected child abuse reported by at least 2 radiologists in your hospital? *

Mark only one oval.

- Yes
- No

If you are using your smartphone, please rotate the screen to landscape

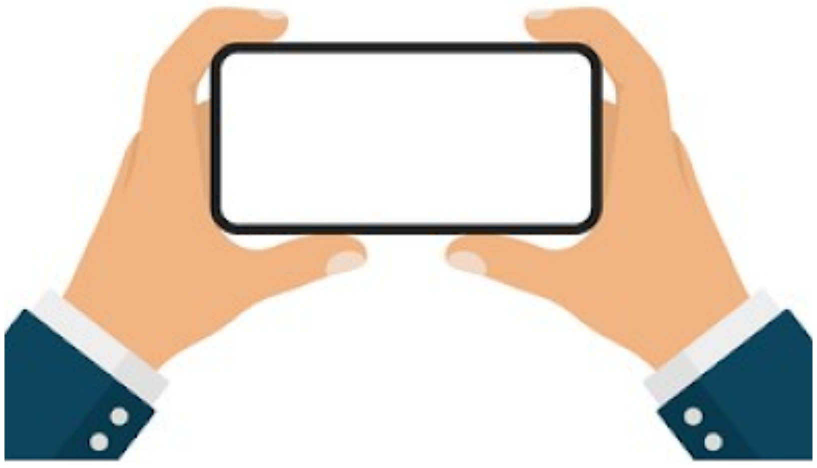

24. To what extent do you agree or disagree with the following statements *

Mark only one oval per row.

	Strongly disagree	Disagree	Neutral	Agree	Strongly agree
**I have the confidence to handle suspected physical abuse in children**					
**I am aware of the radiological signs of inflicted injury in children**					
**I need training in radiological reporting of suspected child abuse**					

Knowledge about child abuse reporting procedure

If you are using your smartphone, please rotate the screen to landscape

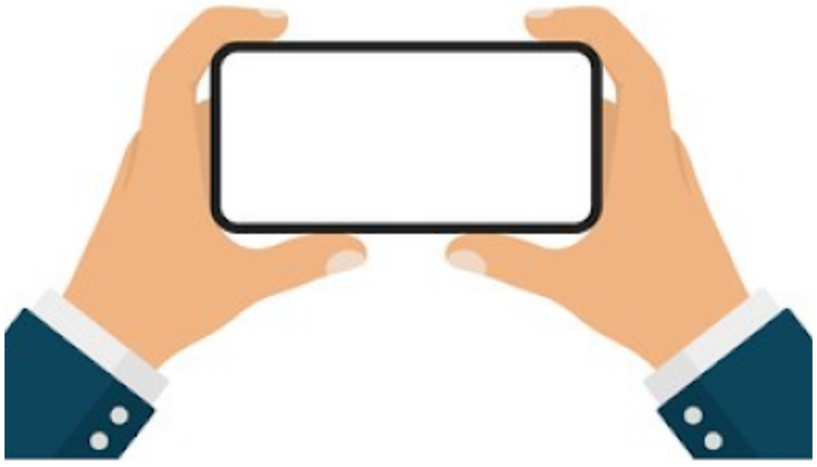

25. To what extent do you agree or disagree with the following statements *

Mark only one oval per row.

	Strongly disagree	Disagree	Neutral	Agree	Strongly agree
**I am familiar with the policy in my hospital for reporting and acting in cases where I suspect child abuse**					
**It is legally mandatory in Saudi Arabia to report child abuse**					
**I am familiar with the National Family Safety Program (NFSP)"**					

26. Additional comments:

This content is neither created nor endorsed by Google.

Google Forms
