# Supplementary material for: Professional practice and awareness of child abuse among radiologists and radiologic technologists: results from Saudi Arabia
Source: Pediatr Radiol. 2022 Dec 15;53(5):832–43. doi: 10.1007/s00247-022-05561-x (PMC10156848; doi:10.1007/s00247-022-05561-x)
Supplement: Supplementary file 3 — Supplementary file3 (DOCX 28.0 KB) [file 247_2022_5561_MOESM3_ESM.docx]

**Online Supplementary Material 3** Respondents’ answers regarding survey questions, with comparisons using Mann-Whitney *U* test

| Variables | Radiologic technologists (*n*=224)  *n* (%) | Radiologists (*n*=91)  *n* (%) |
| --- | --- | --- |
| **Place of work** | | |
| Ministry of Health | 109 (48.7) | 51 (56) |
| Universities’ Hospital | 30 (13.4) | 11 (12.1) |
| National Guard Hospital | 13 (5.8) | 6 (6.6) |
| King Faisal Specialist Hospital & Research Centre | 13 (5.8) | 4 (4.4) |
| Armed Forces Hospital | 16 (7.1) | 6 (6.6) |
| Security Forces Hospital | 11 (4.9) | 3 (3.3) |
| John Hopkins/Aramco Hospital | 4 (1.8) | 0 |
| Royal Commission Hospital | 1 (0.4) | 0 |
| Private Hospital | 27 (12.1) | 10 (11) |
| **I understand the term "inflicted injury" or "physical abuse" or "non-accidental injury” in children?** | | |
| Strongly disagree | 10 (4.5) | 2 (2.2) |
| Disagree | 23 (10.3) | 3 (3.3) |
| Neutral | 37 (16.5) | 1 (1.1) |
| Agree | 106 (47.3) | 31 (34.1) |
| Strongly agree | 48 (21.4) | 54 (59.3) |
| Z=6.61  **(*P*<0.001)^a^** |  |  |
| **Does your practice cover imaging of paediatric patients (children 0 to 18 years)?** | | |
| Yes | 206 (92.0) | 76 (83.5) |
| No | 18 (8.0) | 15 (16.5) |
| **If you answered "Yes", then approximately how many children with suspected physical abuse do you image/report per year?^b^** | | |
| 1–10 | 138/206 (66.9) | 43/76 (56.6) |
| 11–20 | 37/206 (17.9) | 21/76 (27.6) |
| 21–30 | 13/206 (6.3) | 4/76 (5.3) |
| 31–40 | 4/206 (2.0) | 0 |
| 41–50 | 4/206 (2.0) | 1/76 (1.3) |
| 51–60 | 0 | 1/76 (1.3) |
| 61–70 | 2/206 (1.0) | 0 |
| 71–80 | 1/206 (0.5) | 0 |
| 81–90 | 0 | 0 |
| 91–100 | 0 | 0 |
| >100 | 3/206 (1.4) | 2/76 (2.6) |
| Blank | 4/206 (2.0) | 4/76 (5.3) |
| **If you answered "Yes", which guidelines are you aware of?^c^** | | |
| ACR-SPR | 21/31 (67.8) | 29/36 (80.6) |
| RCR-SCoR | 6/31 (19.3) | 4/36 (11.1) |
| ACR-SPR and RCR-SCoR | 4/31 (12.9) | 3/36 (8.3) |
| Others | 0/31 | 0/36 |
| **If you answered "Yes", did the training discuss child injuries related to suspected physical abuse?^d^** | | |
| Yes | 19/59 (32.2) | N/A |
| No | 38/59 (64.4) | N/A |
| Blank | 2/59 (3.4) | N/A |
| **What type of training did you receive?^e^** | | |
| International conference, course or workshop | 4/59 (6.8) | 8/18 (44.4) |
| Local conference, course or workshop | 37/59 (62.7) | 6/18 (33.3) |
| Other: clinical fellowship | 1/59 (1.7) | 0 |
| Other: verbal from the seniors | 1/59 (1.7) | 0 |
| Other: workplace mentoring | 1/59 (1.7) | 0 |
| Others: sessions during residency | N/A | 2/18 (11.1) |
| Blank | 15/59 (25.4) | 2/18 (11.1) |
| **If you answered "Yes", what is the protocol?^f^** | | |
| - AP and lateral skull including cervical spine, AP chest, abdomen and pelvis, including thoracolumbar spine, lateral thoracolumbar spine, AP both upper limbs, bilateral both lower limbs, both feet, both hands | | |
| - Child abuse skeletal X-survey and some cases need CT and MRI | | |
| - Chest and skull radiograph | | |
| - Images for each limb + skull + chest + abdomen + pelvis + spine | | |
| - Skeletal survey, CT and MRI brain | | |
| - Skeletal survey, CT head and abdominal US | | |
| - Skeletal survey | | |
| - Skeletal radiograph, abdominal US | | |
| - Two views for each organ: skull, spine, chest, and one view for pelvis and upper and lower limbs | | |
| - Radiography of skull, limbs, abdomen, chest | | |
| - Radiography AP and lateral skull, AP and lateral chest, two views of extremity | | |
| - Skeletal survey, one exposure only for the whole skeleton | | |

*ACR* American College of Radiology, *AP* anteroposterior, *N/A* not applicable, *RCR* Royal College of Radiologists, *SCoR* Society and College of Radiographers, *SPR* Society for Pediatric Radiology,

^a^ Denotes statistical significance (*P*<0.05), in bold

^b^ For those who answered “Yes” to the following question: “*Does your practice cover imaging of paediatric patients (children 0 to 18 years)?*”

^c^ For those who answered “Yes” to the following question: “*Are you aware of the international guidelines for imaging children with suspected physical abuse?*”

^d^ For radiologic technologists who answered “Yes” to the following question: “*Have you received training (i.e. courses, workshops, workplace mentoring, etc.) in paediatric radiology?*”

^e^ For radiologic technologists and radiologists who answered “Yes”, respectively, to the following questions: “*Have you received training (i.e. courses, workshops, workplace mentoring, etc.) in paediatric radiology?*” and “*Have you received training (i.e. courses, workshops, etc.) in evaluating injury in children related to suspected physical abuse?*”

^f^ For radiologic technologists and radiologists who answered “Yes” to the following question: “*Is there an imaging protocol (i.e. skeletal survey) at your hospital for children younger than 2 years old with suspected physical abuse?*”
